# The Academy of Medicine and the Homœopathists

**Published:** 1867-12

**Authors:** 


### The Academy of Medicine and the Homœopathists.

*To the Editors of the Evening Post:*

The attitude of the Academy of Medicine towards homœopathy and every other exclusive system of medicine is very much misunderstood by the public. The regular school admits most distinctly that every remedy may be beneficial against some one or more diseases; and that it is the duty of the true physician to make himself acquainted with as many curative procedures as possible. The profession is broadly catholic, and accepts improvements from all quarters; but it is also necessarily somewhat conservative, and will not hastily abandon approved methods until it is sure that better have been found. It also imperatively demands that all so-called improvements shall be in accordance with common sense and good judgment; and while it cordially welcomes every real advance in every department of medicine and surgery, it determinedly resists all extremely revolutionary and completely subversive systems.

Thus it must reject the extreme homœopathist with his one law of treatment and his excessively minute doses. For it knows that the law of similarities is only partial, or even only an apparent truth; because a similar thing *differs* somewhat, as well as resembles a great deal; hence, a so-called homœopathic remedy acts somewhat different from, or really allopathically to the disease it is given to cure. In the regular school, remedies are given which act either similarly, differently, or antagonistically to the action of the disease, just as experience and reason require; for this is merely giving medicines which act slightly, greatly, or extremely different from the morbid action. It has no objection to the law of similarities as a partial truth, but rejects homœopathy when it claims to be the only true system of medicine. It rejects infinitessimal doses because they are not only irrational in themselves, but are rejected by the major part of the homœopathists also. The more rational of the homœopathists not only use doses which are not homœopathic, but they are also often obliged to fall back upon the remedies of the regular school in many cases of severe suffering and great danger. Hence, as many of the homœopathists use their medicine in an allopathic way, and use allopathic remedies besides, they must be rejected as long as they publicly claim that homœopathy is an universally true system of medicine. They are simply regarded as recreant allopathists. They get all their knowledge of anatomy, physiology, surgery, midwifery, etc., etc., from the regular school, and a large portion of their materia medica and therapeutics, and merely use a few new or old remedies in a peculiar way. Whenever they condescend to use their own remedies in a rational and professional way, and give their allopathic doses and remedies without mystery or concealment, there will be no quarrel with the Academy of Medicine. Every new remedy which they may discover, every old remedy which they may use in novel and useful ways, will be honestly and carefully tested. The regular school already use many of the so-called homœopathic remedies far more scientifically and wisely than the homœopathists themselves. There is no illiberality against the use of any medicine which is brought forward in a frank, rational and professional way. There is the largest and broadest freedom for the use of any and every remedy which has simple and rational experience in its favor. P.

